# Reply to Garnemark et al. The Swedish National Formulary for Children ePed. Comment on “Shaniv et al. A Callout for International Collaboration. Reply to Giger, E.V.; Tilen, R. Comment on “Shaniv et al. Neonatal Drug Formularies—A Global Scope. *Children* 2023, *10*, 848”. *Children* 2023, *10*, 1803”

**DOI:** 10.3390/children11111341

**Published:** 2024-10-31

**Authors:** Dotan Shaniv, Anne Smits, Karel Allegaert, on behalf of the Neonatal Drug Formularies Group

**Affiliations:** 1 Pharmacy Services, Kaplan Medical Center (Clalit Health Services), Pasternak St., P.O. Box 1, Rehovot 76100, Israel; dotan.shaniv@mail.huji.ac.il; 2 Neonatal Intensive Care Unit, Kaplan Medical Center (Clalit Health Services), Pasternak St., P.O. Box 1, Rehovot 76100, Israel; 3 Department of Development and Regeneration, KU Leuven, 3000 Leuven, Belgium; anne.smits@uzleuven.be; 4 Neonatal Intensive Care Unit, University Hospitals Leuven, 3000 Leuven, Belgium; 5 Department of Pharmaceutical and Pharmacological Sciences, KU Leuven, 3000 Leuven, Belgium; 6 Department of Hospital Pharmacy, Erasmus MC, 3015 GD Rotterdam, The Netherlands

We received the comment from our Swedish colleagues and have read it with interest [1]. First and foremost, we would like to express our gratitude for heeding our call [2] and appreciation for the time and effort invested in authoring this comment, which inducts the Swedish formulary into our constantly growing list of neonatal formularies.

We hereby explicitly value the reporting on their practices and workflows, as this provides additional transparency on the similarities and dissimilarities in practices between the neonatal formularies. Based on this and former comments, we have generated an updated list of neonatal formularies reported in the initial publication by adding the formularies previously unknown to us (see Table 1) [1,2,3].

It is not surprising that the Swedish National Formulary for Children ePed, which currently provides drug information for the entire pediatric age range, started as a database for drug information for neonates; the unique needs of this population and the scarcity of licensed preparations for neonates are well known and have been described in our original paper [3], the comments that followed [2,4], and elsewhere [5,6].

It was interesting to learn that ePed is not only a “classical” formulary in the sense of a repository of data, which requires the clinician to actively reach for information on a certain drug following a clinical question, but it also serves as a decision support tool that is integrated into the electronic health records (EHR) used in Sweden. Such versatility is expected to increase accessibility to drug information and decrease medication errors, which are common in neonatal inpatient settings and may have dire consequences in this fragile population [7,8,9]. Such an elucidation of uses and implementations of formularies also meet the objective of our original publication, as sharing strategies and methods may provide a pathway to implement similar strategies elsewhere, based on the legal, operational, and regulatory settings in a given country or territory.

We were also impressed by the structured approach towards the expansion and maintenance of the formulary. This two-level process involving the regional ePed committees and the national ePed committee ensures a robust and thorough process for developing reliable drug information. The process of updates to the formulary was also well-described and confers an impression of a “reactive” and up-to-date formulary.

As described before, accessibility to reliable drug information is an absolute necessity for any healthcare professional involved in neonatal care. Government-funding hereby allows formularies to be freely accessible, thus enabling them to serve anyone, which is particularly significant for underfunded medical institutions and developing countries. Therefore, the fact that ePed is government-funded and freely accessible is a great advantage. Another aspect of accessibility is the formulary language; as most encountered formularies are country- or region-specific, it is understandable that they are provided in the local language. Obviously, this will likely preclude professionals from outside the region from using the formulary. We were happy to learn that the translation of ePed to English is being considered and discussed, as this will surely contribute greatly to the neonatal community worldwide.

Finally, we would like to repeat our appreciation and gratitude to all groups and authors for taking the time to describe their formularies for the benefit of the readership of the journal. We hereby repeat our call for international collaboration and invite all formularies around the world to join this effort and share their knowledge and methods for the benefit of neonates anywhere—in the absence of labeled indications and doses for neonates, formularies remain crucial to provide evidence-based guidance to healthcare professionals involved in neonatal pharmacology.

## Figures and Tables

**Table 1:** Descriptive characteristics of included neonatal formularies [1,2,3].

#	Name	Publisher	Country	Language	Format	Access	Latest Edition/Year of Printed Publication	URL/ISBN
1	Australasian Neonatal Medicines Formulary (ANMF)	Australasian Neonatal Medicines Formulary (ANMF) consensus group	Australia and New Zealand	English	Digital and print	Public		https://www.anmfonline.org/ (accessed on 19 September 2024)
2	British National Formulary for Children (BNF for Children)	BMJ, Pharmaceutical Press and RCPCH Publications Ltd.	United Kingdom	English	Digital and print	Public (UK) subscription (elsewhere)	2024–2025	9780857114792 https://bnfc.nice.org.uk/ (UK only access for the UK health service) (accessed on 19 September 2024) Worldwide access is available through a subscription to MedicinesComplete: https://about.medicinescomplete.com/ (accessed on 19 September 2024)
3	Dutch/German/Austrian and Norwegian Pediatric Formularies (DPF, also known as “Kinderformularium”)	Foundation Dutch Knowledge Center Pharmacotherapy for Children and Kinderformularium BV	The Netherlands, together with Norway, Germany, and Austria	Dutch German Norwegian	Digital	Public		https://www.kinderformularium.nl/ www.kinderformularium.de www.koble.info www.kindermedika.at (all accessed on 19 September 2024)
4	Pediatric and Neonatal Lexi-Drugs (Lexicomp)	Wolters Kluwer^®^	USA	English (online interface is available in 18 languages)	Digital (web-based and mobile application) and print	Subscription	30th Edition, 2023	9781591953913 https://online.lexi.com/lco/action/home (accessed on 19 September 2024)
5	NeoFax	Merative	USA	English	Digital (web-based and mobile application)	Subscription		https://www.micromedexsolutions.com/home/dispatch/ (accessed on 19 September 2024)
6	Neonatal Formulary: Drug Use in Pregnancy and the First Year of Life	Oxford University Press	United Kingdom	English	Digital and print	Full online version requires subscription	8th Edition, 2020	9780198840787 Supplementary material for the print version is freely available at: https://academic.oup.com/book/35484#login-purchase
7	Neonatal Dosage and Practical Guidelines Handbook	Saudi Ministry of Health	Saudi Arabia	English	Print	Public	2nd Edition, 2016	9786038144848 (accessed on 19 September 2024) Freely available through ResearchGate: https://www.researchgate.net/publication/316235370_Neonatal_Dosage_and_Practical_Guidelines_Handbook (accessed on 19 September 2024)
8	SwissPedDose	Swiss Society of Neonatology	Switzerland	German, French, English, Italian	Digital	Public		https://db.swisspeddose.ch/ (accessed on 19 September 2024)
9	NeoFarma SIBEN	The Ibero-American Society of Neonatology	The Ibero-American countries	Spanish	Digital	Subscription	2nd Edition, 2019	https://siben.net/app/login (accessed on 19 September 2024)
10	The Swedish National Formulary for Children ePed	Astrid Lindgren Children’s Hospital at Karolinska University Hospital	Sweden	Swedish	Digital	Public		https://eped.se/ (accessed on 19 September 2024)
